# Nicardipine Inhibits Breast Cancer Migration *via* Nrf2/HO-1 Axis and Matrix Metalloproteinase-9 Regulation

**DOI:** 10.3389/fphar.2021.710978

**Published:** 2021-08-13

**Authors:** Yen-Chang Chen, Jia-Hong Chen, Cheng-Fang Tsai, Chen-Teng Wu, Miao-Hsiang Wu, Pei-Chun Chang, Wei-Lan Yeh

**Affiliations:** ^1^ Department of Biological Science and Technology, China Medical University, Taichung, Taiwan; ^2^ Institute of New Drug Development, China Medical University, Taichung, Taiwan; ^3^ Department of General Surgery, Taichung Tzu Chi Hospital, Buddhist Tzu Chi Medical Foundation, Taichung, Taiwan; ^4^ Department of Medical Laboratory Science and Biotechnology, Asia University, Taichung, Taiwan; ^5^ Department of Surgery, China Medical University Hospital, Taichung, Taiwan; ^6^ Department of Bioinformatics and Medical Engineering, Asia University, Taichung, Taiwan; ^7^ Department of Biochemistry, School of Medicine, China Medical University, Taichung, Taiwan

**Keywords:** nicardipine, matrix metalloproteinase, heme oxygenase, breast cancer, migration

## Abstract

**Background:** Metastasis represents an advanced stage of cancers, and matrix metalloproteinases are critical regulators. Calcium signal is crucial for appropriate cell behaviors. The efficacy and effects of calcium channel blockers in treating cancers are individually differ from each other. Here, we attempt to investigate the effects of nicardipine, a FDA-approved calcium channel blocker, in advanced breast cancers.

**Methods:** We analyzed the influence of nicardipine on the colony-forming ability of triple negative breast cancer cell lines. Using cell culture inserts, cell migration was also examined. The expression of regulatory proteins was evaluated by real-time PCR, Western blot, and ELISA.

**Results:** We have confirmed that nicardipine inhibits the breast cancer cells migration and colony formation. In addition, we also revealed that nicardipine increases the Nrf2 and HO-1 expression. The inhibition of HO-1 abrogates nicardipine-reduced matrix metalloproteinase-9 expression. Moreover, the end products of HO-1, namely, CO, Fe2+, and biliverdin (will converted to bilirubin), also decreases the expression of matrix metalloproteinase-9.

**Conclusion:** These findings suggest that nicardipine-mediated matrix metalloproteinase-9 reduction is regulated by Nrf2/HO-1 axis and its catalytic end products. Therefore, nicardipine may be a potential candidate for repurposing against advanced breast cancers.

## Introduction

Metastasis is a multistep process representing an advanced stage of malignancy and is the leading cause of cancer-related deaths. One key aspect of metastasis is the invasiveness of the cancer cells, which is mainly driven by cell motility and colony formation (Tahtamouni et al., 2019). In addition, these processes require the involvement of a wide array of molecular alterations such as the expression of proteolytic enzymes (Novikov et al., 2021). The proteolytic activity of matrix metalloproteinases (MMPs) degrades the extracellular matrix (ECM), thereby creating paths for cell migration. MMPs are presented in a variety of human cancers; they are expressed not only by healthy fibroblasts in the adjacent stroma and cancer-associated fibroblasts but also by non-fibroblastic cancer cells (Cathcart et al., 2015). MMPs influence tumor microenvironment by facilitating tumor growth, angiogenesis, and metastasis (Das et al., 2017). In consequence, the MMPs expression is tied to cancer aggressiveness and patient prognosis.

Heme oxygenase (HO) is an inducible enzyme that catalyzes the degradation of heme and produces carbon monoxide (CO), free iron, and biliverdin, which is catabolized into bilirubin (Drummond et al., 2019). Among different HO isoforms, HO-1 is an inducible form which can be stimulated by heme, heavy metals, oxidants, UV irradiation, and inflammatory cytokines (Chau, 2015). In recent years, increasing evidence supports the involvement of HO-1 in several malignant diseases; however, its dual role in cancers is still controversial (Nitti et al., 2017; Chiang et al., 2018; Gandini et al., 2019).

Nicardipine is a dihydropyridine class L-type voltage-gated calcium channels (VGCCs) antagonist used in the treatment of vascular disorders. It has been revealed that L-type calcium channels are frequently altered in different cancer types (Cerami et al., 2012; Gao et al., 2013), and altered L-type calcium channels in breast cancer patients correlate with poor prognosis, by analyzing Oncomine data sets (Rhodes and Chinnaiyan, 2005; Wang et al., 2015; Phan et al., 2017). Antagonizing L-type calcium channels arrests cell cycle and exhibits inhibitory effects on cancer progression (Strobl et al., 1990; Yoshida et al., 2007; Wu et al., 2020). As the inhibition of VGCCs are widely used in clinical practice against cardiovascular or nervous system diseases, increasing studies are targeting VGCCs as cancer therapy (Kale et al., 2015). The accumulating evidence raises hope to look for a possibility of repurposing FDA-approved calcium channel blockers for cancer treatment.

The upstream molecules that regulate MMPs expression or their proteolytic activity are critical in treating cancer metastasis. In the current study, we addressed that nicardipine exerts the antitumor effect by inhibiting the MMP expression and cell migration in breast cancers.

## Results

### Nicardipine Inhibits Cell Migration and Colony Formation in Breast Cancer Cells

**FIGURE 1:**
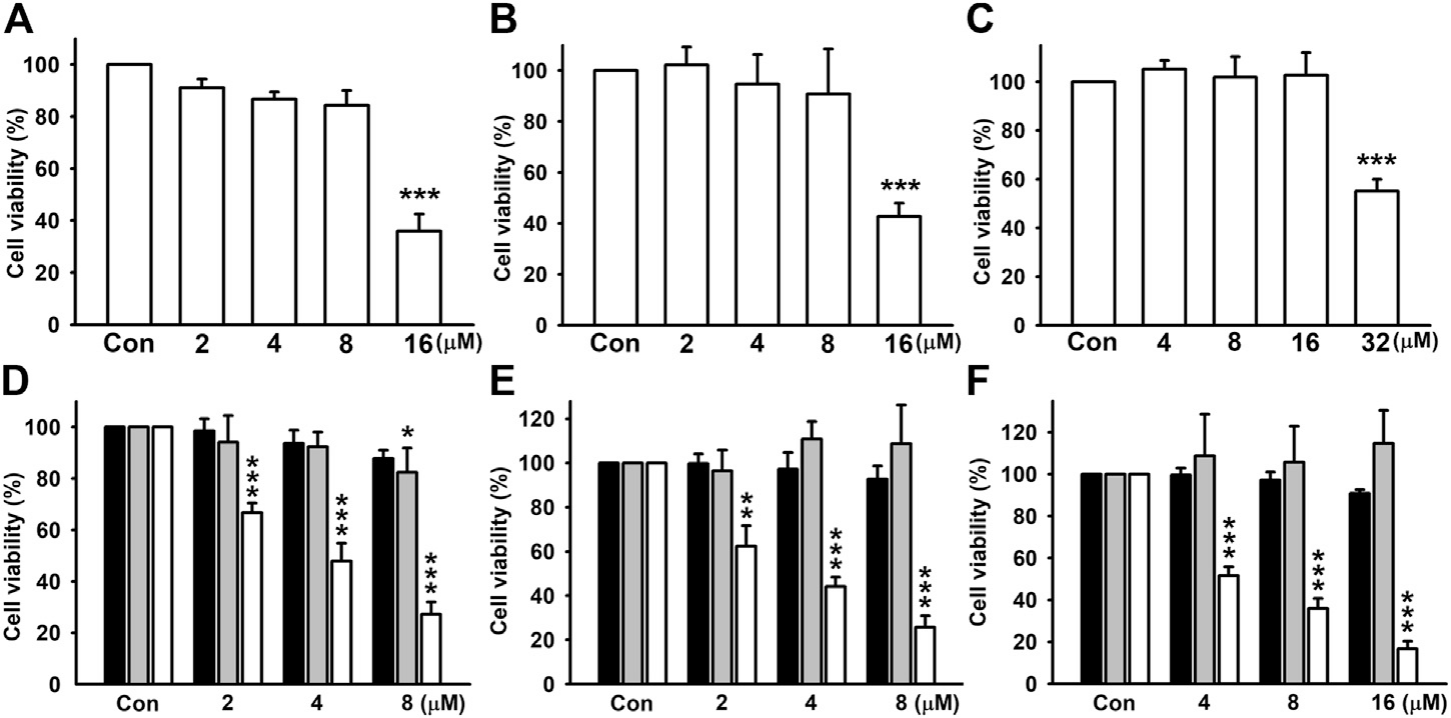
Examination of cell viability of breast cancer cells after nicardipine treatment**.** Examined by the SRB assay, nicardipine given for 24 h caused nonspecific cell death in MDA-MB-231 **(A)**, 4T1 **(B)**, and JC **(C)** breast cancer cells at the highest dosages. The second high dosages were used and administered for 24, 48, or 72 h, and cell viability was evaluated by MTT assay in MDA-MB-231 **(D)**, 4T1 **(E**), and JC **(F)** breast cancer cells (black bar, 24 h; gray bar, 48 h; white bar, 72 h). Graphs showed mean ± S.D. of at least three independent experiments.

Triple-negative breast cancer (TNBC) is associated with a higher metastatic potential and shorter median time to relapse and death than other breast cancer subtypes (Hudis and Gianni, 2011). First, we examined whether the dosages used in this study influence cell viability in all cell lines. By treating cells with different doses of nicardipine for 24 h, cell viability was examined by SRB assays. As shown in Figures 1A–C, the highest dosages significantly caused cell death in MDA-MB-231, 4T1, and JC breast cancer cells; hence, the second high dosages were used in the following experiments. In addition, cell viability evaluated by the MTT assay under indicated treatment of nicardipine for 24, 48, and 72 h is also shown in Figures 1D,E. Note that the dosages of nicardipine used in our study decreased cell viability at 72-h treatment in MDA-MB-231, 4T1, and JC cells. Since nicardipine was reconstituted with DMSO, cells of the control group were treated with 0.1% DMSO as vehicle control (same dilution factor as a nicardipine-treated group).

**FIGURE 2:**
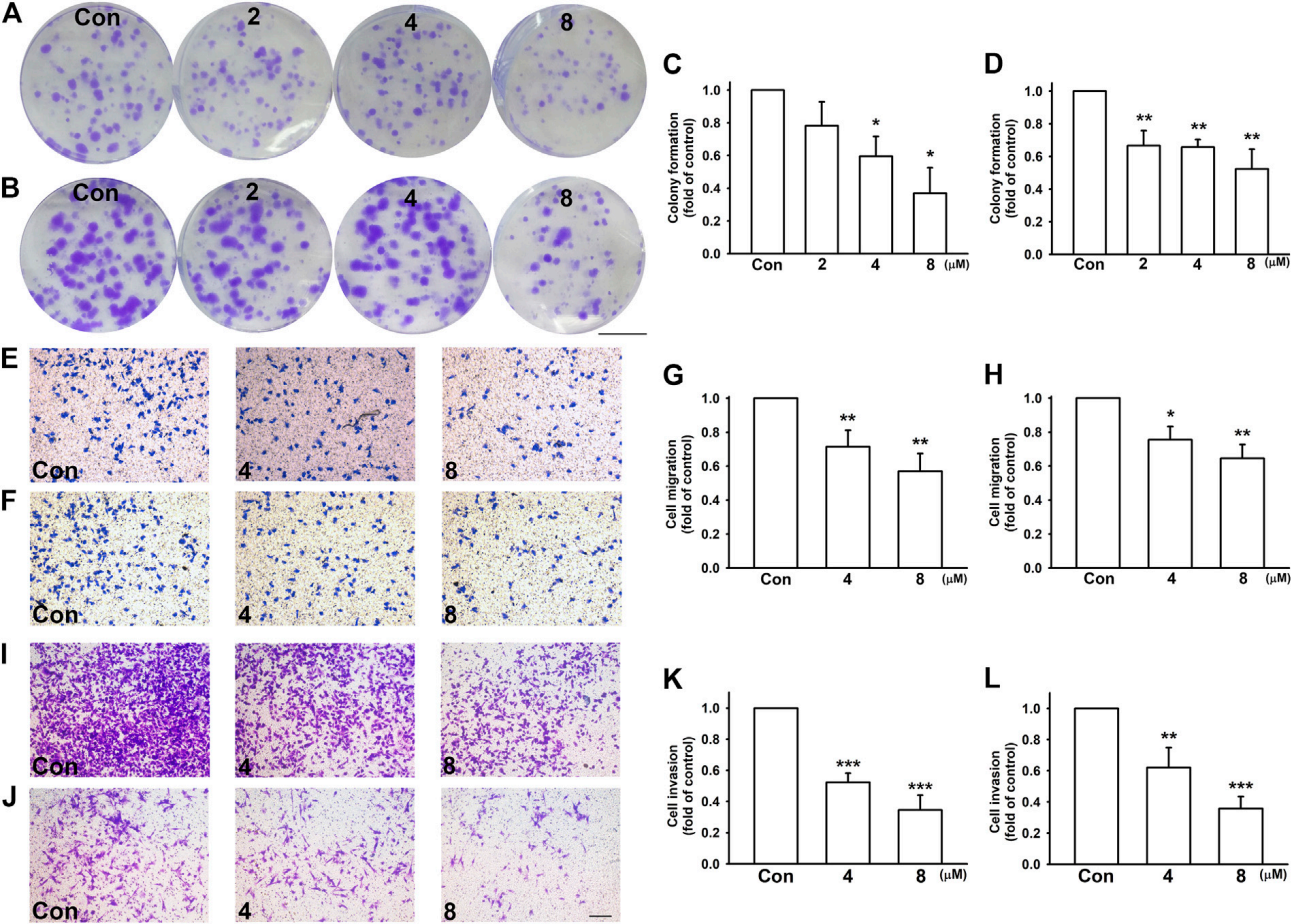
Nicardipine dose dependently decreased colony formation, cell migration, and invasion on breast cancer cells. Cells were treated by indicated concentrations of nicardipine for 10 days, and the medium and administration of nicardipine were refreshed every other day. Formed colonies were stained by crystal violet, and colonies larger than 1-mm diameter were counted. Scale bar, 1 cm. Note that nicardipine reduced colony formation in both MDA-MB-231 cells **(A,C)** and 4T1 cells **(B,D)**. For cell migration and invasion assay, cells seeded in transwells were treated with different dosages of nicardipine, and cells migrated to the other side of the filter were stained by crystal violet and counted. Cell migration ability of both MDA-MB-231 **(E,G)** and 4T1 **(F,H)** were downregulated by nicardipine dose dependently. Cell invasion ability of both MDA-MB-231 **(I,K)** and JC **(J,L)** were also dose dependently downregulated. Scale bar, ×100 magnification. Graphs showed mean ± S.D. of at least three independent experiments. *p < 0.05; **p < 0.01 compared to control group. Nicardipine reduces MMP-9 but not MMP-2 expressions in breast cancer cells.

To investigate the effect of nicardipine on colony formation, the cells were seeded at a low density, and gradually formed colonies were examined 10 days later. Nicardipine markedly inhibited colony formation in a dose-dependent manner in both MDA-MB-231 and 4T1 breast cancer cells (Figures 2A,B). Compared with control, the colony-forming ability was reduced to 0.37 ± 0.16-fold and 0.52 ± 0.12-fold in MDA-MB-231 and 4T1 cells, respectively (Figures 2C,D). For testing the ability of cell migration, breast cancer cells were treated with different concentrations of nicardipine and cell motility was analyzed by cell culture inserts. At the highest dosage of 8 μM, nicardipine notably inhibited cell migration to 0.57 ± 0.10-fold and 0.65 ± 0.08-fold of control in MDA-MB-231 (Figures 2C,D) and 4T1cells (Figures 2F,H), respectively. Similarly, 8 μM nicardipine markedly abrogated cell invasion to 0.35 ± 0.09-fold and 0.36 ± 0.08-fold of control in MDA-MB-231 (Figures 2I,K) and JC (Figures 2J,L) cells, respectively. These results suggest that nicardipine exerts significant effects on inhibiting the cell motility and colony-forming ability in triple negative breast cancer cells.

### Nicardipine Reduces MMP-9 But Not MMP-2 Expressions in Breast Cancer Cells

**FIGURE 3:**
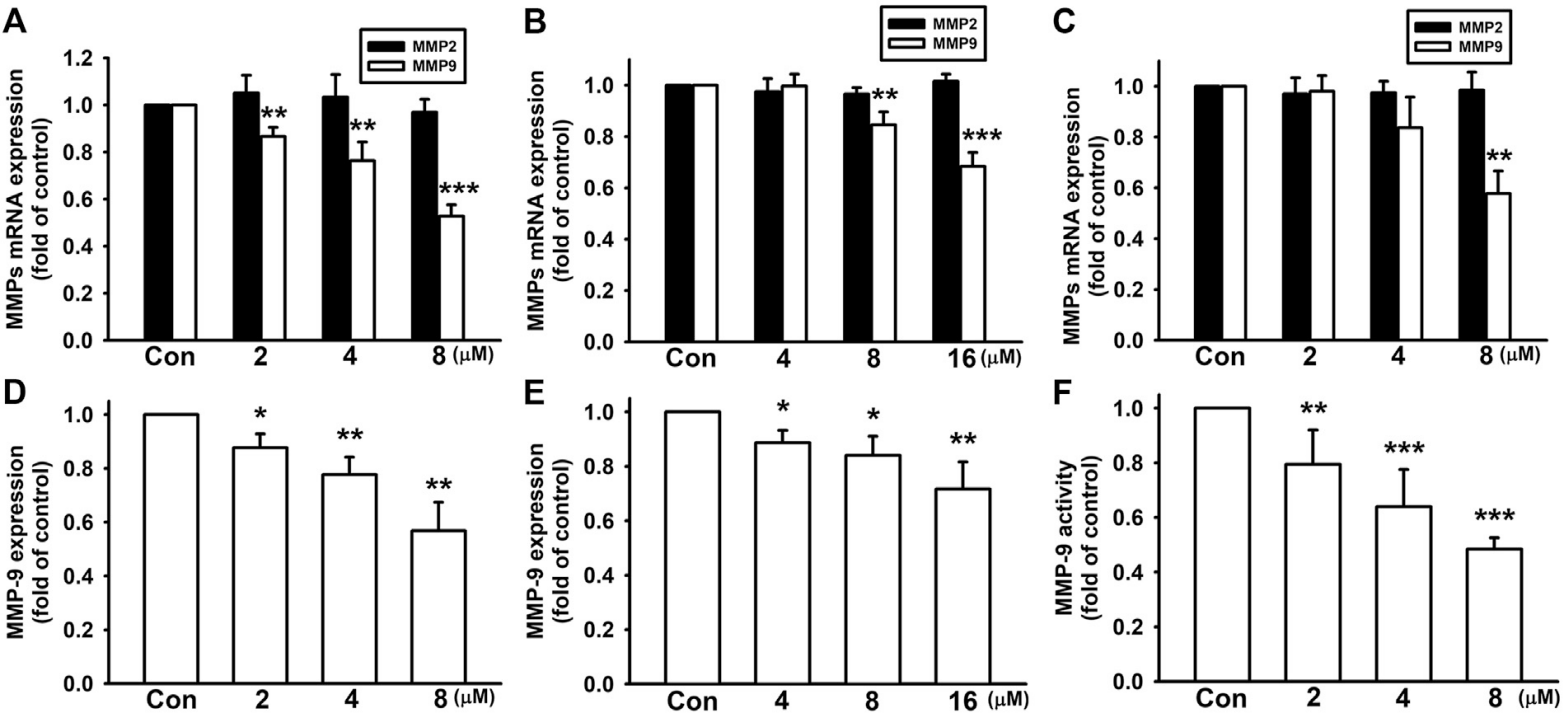
Nicardipine decreased MMP-9 expression in breast cancer cells. After treati-ng cells with nicardipine for 6 h, we found that nicardipine dose dependently reduced MMP-9 mRNA expression but not MMP-2 expression on 4T1 cells **(A)**, JC cells **(B)**, and MDA-MB-231 cells **(C)**. Analyzed by MMP-9 ELISA, nicardipine (24 h) also reduced MMP-9 expression in 4T1 **(D)** and JC **(E)** cells in a dose-dependent manner **(F)** Examined by zymography, nicardipine (24 h) also dose dependently decreased MMP-9 activity in MDA-MB-231 cells. Graphs showed mean ± S.D. of at least three independent experiments. *p < 0.05; **p < 0.01; ***p < 0.001 compared to the control group. Nicardipine elevates HO-1 expression in breast cancer cells.

Among different MMPs, MMP-2 (gelatinase-A) and MMP-9 (gelatinase-B) are strongly expressed and correlated with the tumor invasion and metastasis in breast cancer cells (Farina and Mackay, 2014; Raeeszadeh-Sarmazdeh et al., 2020). Here, we examined whether nicardipine affect MMP expressions in breast cancer cells. Analyzed by quantitative real-time PCR, nicardipine decreased MMP-9 mRNA expression in a dose-dependent manner; however, MMP-2 expression was not affected by nicardipine in 4T1, JC, and MDA-MB-231 breast cancer cells (Figures 3A–C). At the highest dosages of nicardipine treated on 4T1, JC, and MDA-MB-231 cells, the MMP-9 mRNA expression was decreased to 0.53 ± 0.05-fold, 0.68 ± 0.05-fold, and 0.58 ± 0.09-fold of control, respectively. We further analyzed MMP-9 expression by mouse MMP-9 ELISA, and found that nicardipine also dose dependently reduced the protein expression of MMP-9 on 4T1 and JC cells to 0.57 ± 0.11-fold and 0.72 ± 0.10-fold of control at the highest dosages, respectively (Figures 3D,E). Moreover, MMP-9 enzymatic activity of MDA-MB-231 cells was alternatively analyzed by zymography. As shown in Figure 3F, MMP-9 activity was also reduced to 0.48 ± 0.04-fold of control at the highest dosage treated on MDA-MB-231 cells. These results indicated that nicardipine significantly reduced the MMP-9 expression and activity in breast cancer cells.

### Nicardipine Elevates HO-1 Expression in Breast Cancer Cells

**FIGURE 4:**
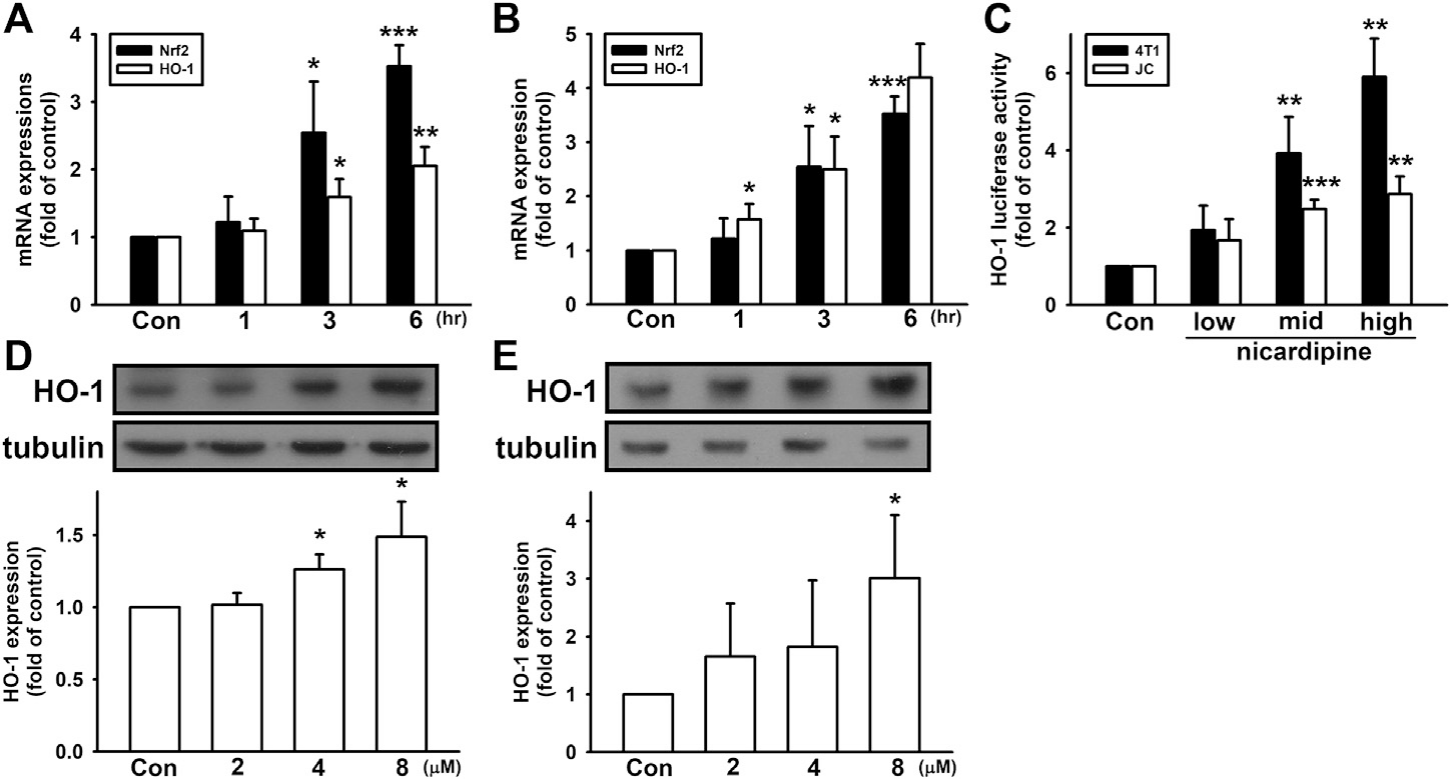
Nicardipine enhanced Nrf2 and HO-1 expression in breast cancer cells. Nicardipine (8 µM) time-dependently induced both Nrf2 and HO-1 mRNA expressions in MDA-MB-231 **(A)** and 4T1 cells **(B)**. **(C)** Examined by luciferase reporter assay, HO-1 expression was also elevated by nicardipine in 4T1 and JC cells. Nicardipine (24 h) increased HO-1 protein expression in a dose-dependent manner in MDA-MB-231 **(D)** and 4T1 cells **(E).** Graphs showed mean ± S.D. of at least three independent experiments. *p < 0.05; **p < 0.01; ***p < 0.001 compared to the control group. HO-1 mediates nicardipine-inhibited MMP-9 expression in breast cancer cells.

In our previous study, we showed that MMP expression can be regulated by HO-1 in breast cancer cells (Tsai et al., 2018). Here, we further investigated whether HO-1 is involved in nicardipine-reduced MMP-9 expression. As shown in Figures 4A,B, nicardipine time dependently enhanced mRNA expression of HO-1 on both MDA-MB-231 and 4T1 cells. After treating nicardipine (8 μM) for 6 h, HO-1 mRNA expression was elevated to 2.05 ± 0.28-fold and 4.20 ± 0.62-fold of control on MDA-MB-231 and 4T1 cells, respectively. Nuclear factor erythroid 2–related factor 2 (Nrf2) is a key transcription factor upstream of HO-1. Here, Nrf2 also exhibited a time-dependent increase under the treatment of nicardipine. Compared to control, the Nrf2 mRNA expression was increased to 2.40 ± 0.72-fold and 3.52 ± 0.32-fold at 6 h on MDA-MB-231 and 4T1 cells, respectively. By transfection of HO-1 promoter-luciferase construct, the luciferase activity was also markedly enhanced by treating nicardipine (Figure 4C). By administering nicardipine, 8 μM on 4T1 cells and 16 μM on JC cells, luciferase activity was elevated to 5.90 ± 0.99-fold and 2.86 ± 0.46-fold of control, respectively. Furthermore, nicardipine also dose dependently increased HO-1 protein expression to 1.49 ± 0.24-fold and 3.01 ± 1.09-fold of control at the highest dosage on MDA-MB-231 and 4T1 cells, respectively (Figures 4D,E).

### HO-1 Mediates Nicardipine-Inhibited MMP-9 Expression in Breast Cancer Cells

**FIGURE 5:**
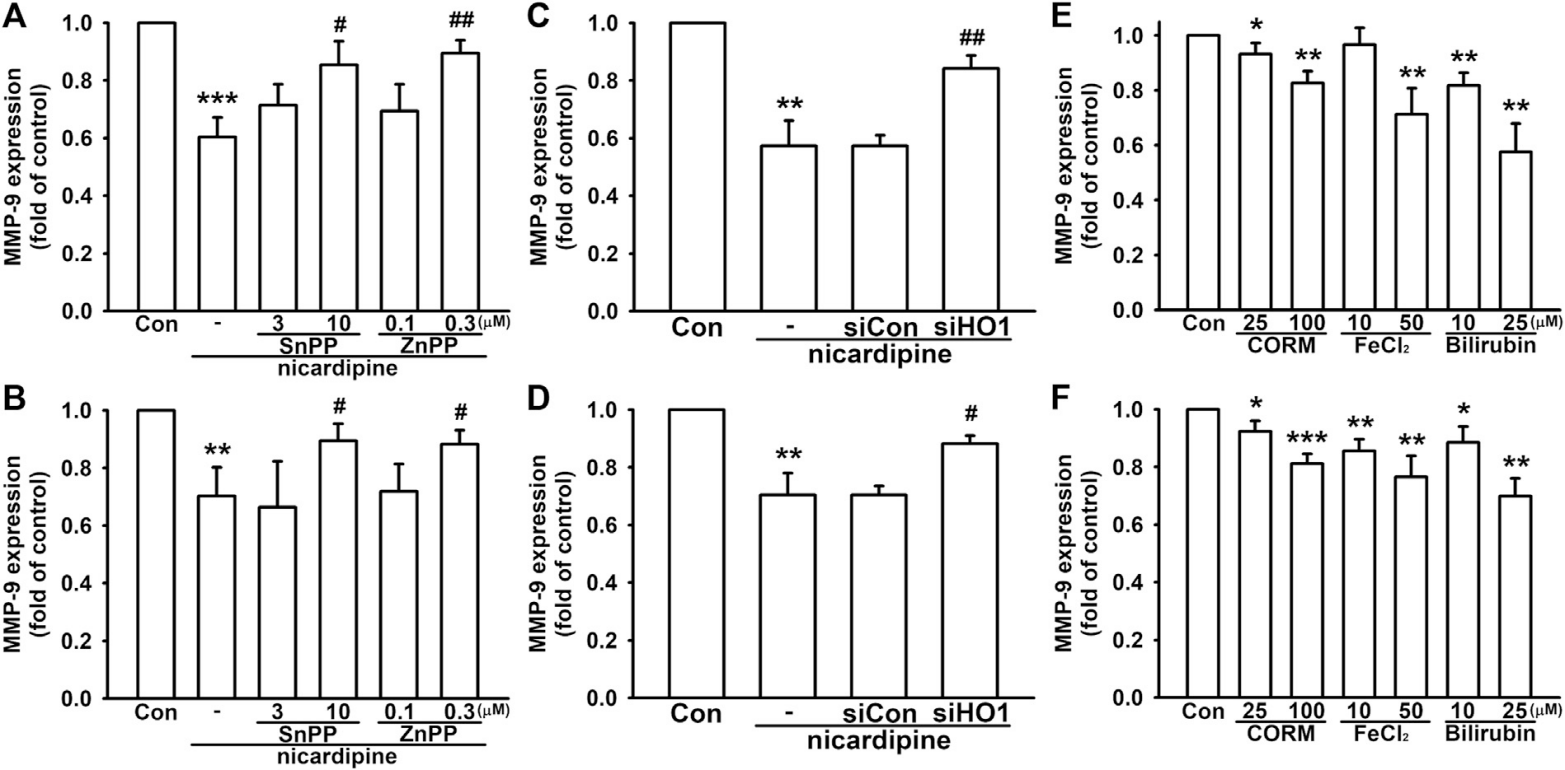
Nicardipine-inhibited MMP-9 expression is regulated by HO-1 in breast cancer cells. Examined by MMP-9 ELISA, inhibition of HO-1 by HO-1 inhibitors, SnPP and ZnPP, dose dependently antagonized nicardipine-decreased MMP-9 expression on 4T1 **(A)** and JC cells **(B)**. Nicardipine-reduced MMP-9 expression was reversed by transfection of HO-1 siRNA, but not influenced by control siRNA on 4T1 **(C)** and JC cells **(D)**. MMP-9 expression was dose dependently decreased by treating HO-1 down-stream effectors, CORM, FeCl2, and bilirubin, on 4T1 **(E)** and JC cells **(F)**. Graphs showed mean ± S.D. of at least three independent experiments. *p < 0.05; **p < 0.01; ***p < 0.001 compared to the control group. #*p* < 0.05; ##*p* < 0.01 compared to the nicardipine-treated group.

In order to confirm the involvement of HO-1 in mediating MMP-9 expression affected by nicardipine, breast cancer cells were subjected to HO-1 inhibition by SnPP and ZnPP. As shown in Figures 5A,B, both SnPP and ZnPP dose dependently reversed nicardipine-inhibited MMP-9 expression on 4T1 and JC cells. Moreover, by transfection of siRNA against HO-1, nicardipine-inhibited MMP-9 expression was also reversed on 4T1 and JC cells. We also confirmed that control siRNA exerted no influence (Figures 5C,D). As HO-1 catalyzes heme into CO, free iron, and biliverdin, which is catabolized into bilirubin (Drummond et al., 2019); hence, we used CORM2, FeCl_2_, and bilirubin to demonstrate the role of HO-1 end products in MMP-9 expression. As shown in Figure 5E, CORM, FeCl_2_, and bilirubin notably reduced the MMP-9 protein expression to 0.83 ± 0.04-fold, 0.71 ± 0.09-fold, and 0.58 ± 0.10-fold of control on 4T1 cells, respectively. Similarly, CORM, FeCl_2_, and bilirubin significantly inhibited the MMP-9 protein expression to 0.81 ± 0.03-fold, 0.77 ± 0.07-fold, and 0.70 ± 0.06-fold of control on JC cells, respectively (Figure 5F). These results indicated that the inhibition of MMP-9 expression by nicardipine is mediated by HO-1 and its downstream effectors in breast cancer cells.

## Discussion

The role of calcium has been documented in various cellular processes, including cell apoptosis, adhesion, motility, and modulation of various intracellular enzymes. Nevertheless, the relationship among intracellular calcium concentration, the use of calcium channel blockers (CCBs) and the risk of cancers is a controversial issue for years. In 1990s, studies indicated that the use of CCBs increases the risk of various cancers (Pahor et al., 1996a; Pahor et al., 1996b; Hardell et al., 1996; Kaplan, 1996). It was proposed that blockage of calcium channels leads to apoptosis inhibition, resulting in a tumor-favoring effect (Durham and Walton, 1982; Trump and Berezesky, 1995). However, a recent large population–based cohort study provided strong evidence that the use of CCBs is not associated with an elevated risk of cancer (Grimaldi-Bensouda et al., 2016; Wilson et al., 2016; Brasky et al., 2017; Raebel et al., 2017), followed by increasing studies attempting to reveal the antitumor effects of CCBs. Yet, the antitumor effects of CCBs still differ from each other. It has been reported that the combination treatment of certain CCBs with chemotherapy induces apoptosis and autophagy of chemoresistant lung cancer (Wong et al., 2020). In addition, verapamil effectively inhibits tumor progression of chemoresistant pancreatic cancer (Zhao et al., 2016). Amlodipine is considered as an inducer of programmed death-ligand 1 (PD-L1) degradation (Li et al., 2020a). It has also been reported that L-type CCBs impairs filopodia formation and cell motility (Jacquemet et al., 2016). In accordance with these studies, our represented study also demonstrated the beneficial anti-cancerous effect against tumor colony-forming and migratory ability.

Nicardipine belongs to the dihydropyridine class of L-type calcium channel blockers and is primarily an arteriolar vasodilator for hypertension and angina. The general oral dosage starts at 20 mg, 3 times a day; however, the dosage ranges from 60 to 120 mg per day. It has been reported that for racemic nicardipine, the linear relationships were found with the serum concentrations of 0.25–80 mg/ml for both enantiomers (Iwaoka et al., 1995). In addition, nicardipine has a volume of distribution of 8.3 L/kg, and the systemic bioavailability is about 35% following a 30-mg oral dose after completely absorbed at a steady state according to the DrugBank database (Wishart et al., 2018). On the other hand, 30 mg/kg oral nicardipine also causes decrease in blood pressure in renal hypertensive rats, deoxycorticosterone acetate/salt hypertensive rats (Takenaka et al., 1985), and spontaneously hypertensive rats (Takenaka et al., 1985; Whiting, 1987). The oral LD_50_ toxicity in rats is 184 mg/kg (Wishart et al., 2018). The dosages we used were in the range of 8–16 µM for different breast cancer cells that equals to 4.13–8.26 μg/ml, which were far below the dosage for antihypertension.

In addition to the role of MMPs in cell motility, epithelial–mesenchymal transition is also an important process that may involve in increased cell motility. The cells lose epithelial cell morphology and reduce the expression of epithelial markers such as α-E-catenin while acquiring mesenchymal-like properties such as increased N-cadherin and β-catenin expressions. However, under the treatment of nicardipine, no significant influence was observed on MDA-MB-231, 4T1, or JC breast cancer cells (Supplementary Figure 2).

The dual role of Nrf2/HO-1 axis in cancers is still under dispute in recent years. It is well-known that Nrf2/HO-1 axis is crucial in cellular adaptation and protection in reducing electrophiles and reactive oxygen species (ROS), thus decreasing DNA damages and decreasing genomic instability (Loboda et al., 2016; Li et al., 2020b). Moreover, some reports demonstrated that HO-1 is elevated in various human malignancies and contributes to settle the tumor microenvironment for cancer cell growth, angiogenesis, and metastasis, as well as resistance to therapy, regarded as a survival molecule exerting a favoring role in cancer progression (Hjortso and Andersen, 2014; Nitti et al., 2017). Interestingly, the upregulated HO-1 expression in tumor tissues may also be further increased in response to therapies (Jozkowicz et al., 2007). In our previous study, we found that HO-1 upregulation induced by fisetin, a dietary flavonoid, inhibits breast cancer migration (Tsai et al., 2018). Similarly, it has also been reported that the overexpression of HO-1 induces apoptosis and cell cycle arrest in breast cancer cell lines and reduces tumor burden in animal model (Gandini et al., 2019). In addition, HO-1 overexpression impedes cancer progression by reducing several onco-microRNAs on hepatocellular carcinoma (Zou et al., 2016). These reports indicate that the role of HO-1 in cancers may be cell type specific and context dependent. In this presented study, we have demonstrated that HO-1 induced by nicardipine exerts antitumor effects by inhibiting cell motility *via* suppressing the MMP-9 expression in breast cancer cells.

In accordance with our previous study (Tsai et al., 2018), accumulating evidence has reported that the HO-1 expression inhibits cancer migration and invasion through decreasing the expression of MMPs (Lin et al., 2008; Chao et al., 2013; Gandini et al., 2019). Among the MMPs, MMP-2 and MMP-9 are strongly correlated with tumor invasion and metastasis in breast cancers (Li et al., 2017), as MMP-2 is more constitutively expressed in metastatic cancers and MMP-9 is highly inducible by various stimuli (Raeeszadeh-Sarmazdeh et al., 2020). Since HO-1 catalyzes heme into CO, Fe^2+^, and biliverdin which converts to bilirubin, we verified the individual role of HO-1 end products in reducing the MMP-9 expression. We found that CO, Fe^2+^, and bilirubin can all markedly decrease MMP-9 expression, while bilirubin was the most significant. CO, as a second messenger, inhibits MMPs has been reported (Tsai et al., 2017). CO may also directly or indirectly involve in the HO-1–reduced MMPs activity (Lin et al., 2008). Similar in other reports, bilirubin progressively and markedly reduced MMP-9 mRNA and protein expression in granulation tissues in diabetic rats (Ram et al., 2016). It has also been reported that 12-O-tetradecanoylphorbol-13-acetate (TPA)–induced MMP-9 expression is also decreased by CO, Fe^3+^, and bilirubin in MCF-7 breast cancer cells (Chao et al., 2013). In addition, it has been reported that CO inhibits the phosphorylation of ERK and c-Jun induced by TPA, which inhibits MMP-9 activity on MCF-7 breast cancer cells (Lin et al., 2008). Moreover, bilirubin also inhibits phosphorylation of p38-MAPK (Iwasa et al., 2003), which mediates cellular mitogenesis and motility (Hamanoue et al., 2016; Naffa et al., 2020). Both biliverdin and bilirubin are potent antioxidants (Gazzin et al., 2016; Seyed Khoei et al., 2020), and the level of ROS positively regulates MMP expression and invasive properties of cancer cells (Mori et al., 2019). However, the effects of HO-1 on cell motility suggest that the phenomenon may not depend on HO-1 alone, but is determined by several players, including CO combined with the antioxidant bilirubin and the sequestration of iron by ferritin could all contribute to suppression of cell motility.

## Conclusion

**FIGURE 6:**
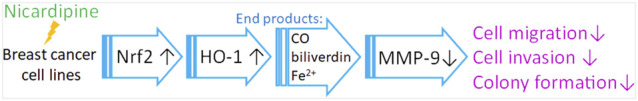
Graphic diagram shows that nicardipine inhibits breast cancer migration *via* Nrf2/HO-1 axis and matrix metalloproteinase-9 regulation.

In conclusion, nicardipine increases Nrf2 expression and sequentially enhances HO-1 expression. The catalytic end products of HO-1 consequently result in MMP-9 inhibition and cell migration in breast cancer cells (Figure 6). Therefore, nicardipine may be a potential candidate for repurposing against advanced breast cancers.

## Materials and Methods

### Materials

Materials used in this study were described in Supplementary File (Supplementary Figure 1).

### Cell Lines

4T1 cell was obtained from American-Type Culture Collection (Manassas, VA) and cultured in an RPMI-1640 Medium (Thermo Fisher Scientific, Waltham, MA). JC cell was obtained from Bioresource Collection and Research Center (Hsinchu, Taiwan) and cultured in the RPMI-1640 medium supplemented with 1 mM sodium pyruvate and 4.5 g/L glucose. MDA-MB-231 cells were obtained from Bioresource Collection and Research Center (Hsinchu, Taiwan) and were cultivated in the Leibovitz’s L-15 medium. Cells were cultured in 37°C incubator with 95% air and 5% CO_2_, except for MDA-MB-231 cells, which was in 95% air without CO_2_. Passages of 10–25 were used in this study.

### Assays for Cell Viability

Cell viability was examined after treating indicated concentrations of nicardipine for 24, 48, or 72 h. For the sulforhodamine B (SRB) colorimetric assay, cells were fixed with trichloroacetic acid (10%) for 10 min and then stained by 0.4% (w/v) SRB in 1% acetic acid for 15 min, followed by washing with 1% acetic acid. After dissolving the cells by Tris solution (10 mM), spectrophotometric quantitation (OD 515 nm) was performed by a SpectraMax M5 plate reader (Molecular Devices, Sunnyvale, CA, United states) (Tsai et al., 2019a).

For MTT assay, the cells were incubated with the MTT [3-(4,5-cimethylthiazol-2-yl)-2,5-diphenyl tetrazolium bromide] solution (0.5 mg/ml in PBS) in 37°C incubator for 1 h. After a brief wash, cells were lysed with DMSO, and spectrophotometric quantitation (OD 550 nm) was performed by a SpectraMax M5 plate reader (Molecular Devices, Sunnyvale, CA, United States) (Tsai et al., 2019b).

### Colony Formation

200 cells were seeded per well in a 6-well plate. After letting adhere overnight, cells were given indicated treatment for 10 days. We also refreshed the medium and added nicardipine on the 3rd, 6^th^, and 9th day. At the end of the treatment, cells were stained with 1% crystal violet solution for 5 min, and the number of colonies was counted with a diameter larger than 1 mm (Tsai et al., 2018).

### Cell Migration

After seeding 1 × 10^4^ cells in 50 μL medium in the upper chamber of cell culture inserts and 500 μL medium in the lower chambers (24-well, 8-μm pore size, Costar New York, NY), cells were let adhere to the membrane for 4 h before indicated treatment. Next, we added 50 μL medium mixed with nicardipine (4 or 8 μM) into the upper chambers. After another 24 h, the cells on the upper side of the membrane were removed, and the cells on the underside were stained by 0.05% crystal violet for 5 min. The chambers were washed with PBS and redundant solution was removed before being photographed (Yeh et al., 2009).

### Cell Invasion

Each cell culture inserts (24-well, 8-μm pore size, Costar New York, NY) were coated 20 μL Corning Matrigel (Corning^®^ Matrigel^®^ Basement Membrane Matrix, catalog number: 354,234) before used. After placing plates in 37°C incubator for 30 min for solidifying the gel, 5 × 10^4^ cells in 50 μL medium were seeded in the upper chambers along with 500 μL medium in the lower chambers. Cells were let adhere to the gel surface for 4 h before indicated treatment. Next, we added 50 μL medium with nicardipine (4 or 8 μM) in the upper chambers. After another 24 (for JC cells) or 48 h (for MDA-MB-231 cells), cells and gel on the upper side of the membrane were removed, and cells on the underside were stained by 0.05% crystal violet for 5 min. The chambers were washed with PBS and the redundant solution was removed before being photographed (Yeh et al., 2009).

### Western Blot

Cells were lysed by the protein extraction solution (Sigma-Aldrich Co., St. Louis, MO, United States) and then placed on ice for 20 min before centrifuged 14,000 rpm for 30 min for protein extraction. The supernatants were collected as whole cell protein samples followed by quantitative analysis using the pierce™ BCA protein analysis kit (Thermo Scientific, Waltham, MA). 25 μg proteins were prepared and boiled at 95°C for 5 min and subjected to SDS-PAGE. After transferring protein to the PVDF membrane (Millipore, Billerica, MA) and blocking with 7.5% skim milk in TBST at room temperature for an hour, the membrane was hybridized with anti–HO-1 antibody (Enzo, Farmingdale, NY) overnight at 4°C. After washing with PBST thrice, the membrane was hybridized with anti-rabbit secondary antibody for 1 h. Protein signals were visualized by enhanced chemiluminescence (EMD Millipore, Billerica, MA) using Fujifilm Super RX-N films (Valhalla, NY), and the intensity of signals was analyzed by ImageJ (Schneider et al., 2012).

### Quantitative Real-Time PCR

Cells were treated with indicated concentrations of nicardipine for 6 h before cell lysis. 10 ng total RNA extracted by TRIzol Reagent (Thermo Fisher Scientific, Waltham, MA) was reverse-transcribed by the cDNA Reverse Transcription Kit (Thermo Fisher Scientific, Waltham, MA) and then subjected to PCR using SYBR Green Master Mix (Applied Biosystem, Singapore). PCR was performed under the following conditions: 95°C for 10 min, 45 cycles at 95°C for 10 s, and then 60°C for 1 min. Primer sequences were described in Supplementary File (Supplementary Figure 1) (Tsai et al., 2019b).

### MMP-9 ELISA Kit

After indicated the treatment of nicardipine for 24 h, culture supernatants were collected and centrifuged at 5,000 rpm for 15 min before subjected to MMP-9 ELISA. Secreted MMP-9 in the supernatant was measured by MMP-9 ELISA kit (Abcam, Cambridge, MA) according to the manufacturers’ protocol.

### Gelatin Zymography

After indicated treatment of nicardipine for 24 h, culture supernatants were collected and centrifuged at 5,000 rpm for 15 min before subjected to SDS-PAGE containing 0.1% gelatin at 4°C. Afterward, gels were washed with 2.5% Triton X-100 (in 50 mM Tris-HCl) and then digested in the developing buffer (50 mM Tris-HCl containing 5 mM CaCl_2_, 1 mM ZnCl_2_, 0.02% NaN_3_, and 1% Triton X-100) at 37°C for 24 h. After staining the gel with Coomassie brilliant blue, the gels were destained in the destaining buffer [50% methanol and 10% acetic acid (v/v)]. The locations of MMPs were detected as clear bands (Tsai et al., 2018).

### Luciferase Reporter Assay

Cells were exposed to the indicated treatment 24 h after transfecting the cells by pHO-1-luciferase plasmid. Afterward, the passive lysis buffer (Promega, Madison, WI) was added followed by shaking the plate for 15 min. 20 µl lysates were transferred into an opaque black 96-well plate, and firefly luciferase activity values were read and normalized by the Renilla luciferase activity (Tsai et al., 2018).

### Statistics

Statistical analysis was performed by using GraphPad Prism. Values are expressed as mean ± S.D. of at least three independent experiments. Results were analyzed with one-way analysis of variance (ANOVA) and significance was defined as *p* < 0.05.

## Data Availability

The original contributions presented in the study are included in the article/Supplementary Material; further inquiries can be directed to the corresponding author.

## Abbreviations

CCBs, calcium channel blockers; CO, carbon monoxide; ECM, extracellular matrix; HO, heme oxygenase; MMP, matrix metalloproteinases; Nrf2, nuclear factor erythroid 2–related factor 2; SRB, sulforhodamine B; TNBC, triple negative breast cancer; VGCC, voltage-gated calcium channel.
