# Supplementary material for: Nicardipine Inhibits Breast Cancer Migration via Nrf2/HO-1 Axis and Matrix Metalloproteinase-9 Regulation
Source: Front Pharmacol. 2021 Aug 13;12:710978. doi: 10.3389/fphar.2021.710978 (PMC8414136; doi:10.3389/fphar.2021.710978)
Supplement: Supplementary file 1 [file DataSheet1.pdf]

Materials	Brand (Catalog Number)
Nicardipine	Cayman (17537), reconstitute in DMSO
HO-1 antibody	Enzo (ADI-SPA-895)
α E-catenin antibody	Santa Cruz (sc-1495)
β-catenin antibody	Abcam (ab16051)
αSMA antibody	Abcam (ab5694)
N-cadherin antibody	Santa Cruz (sc-59987)
α-Tubulin antibody	Sigma-Aldrich (T5168)
β-Actin antibody	Sigma-Aldrich (A5441)
GAPDH antibody	BioLegend (649202)
Anti-mouse antibody	Cell Signaling (#7076)
Anti-rabbit antibody	Cell Signaling (#7074)
MMP9 ELISA kit	Abcam (ab253227)

Primers	Sequences
Human HO-1	F: CCAGGCAGAGAATGCTGAGTTC<br>R: AAGACTGGGCTCTCCTTGTTGC
Mouse HO-1	F: CAGCCCCACCAAGTTCAAAC<br>R: AGGCGGTCTTAGCCTCTTCTG
Mouse MMP-2	F: CGGTTTATTTGGCGGACAGT<br>R: GCCTCATACACAGCGTCAATCTT
Mouse MMP-9	F: CCCTGGAACTCACACGACATC<br>R: TCACACGCCAGAAGAATTTGC
Human Nrf2	F: CACATCCAGTCAGAAACCAGTGG<br>R: GGAATGTCTGCGCCAAAAGCTG
Mouse Nrf2	F: CGAGATATACGCAGGAGAGGTAAGA<br>R: GCTCGACAATGTTCTCCAGCTT
Human 36B4	F: CGACCTGGAAGTCCAACTAC<br>R: ATCTGCTGCATCTGCTTG
Mouse 36B4	F: AGATGCAGCAGATCCGCAT<br>R: GTTCTTGCCCATCAGCACC

Supplementary Figure 1. Materials and primers used in quantitative real time PCR.

Supplementary Figure 2

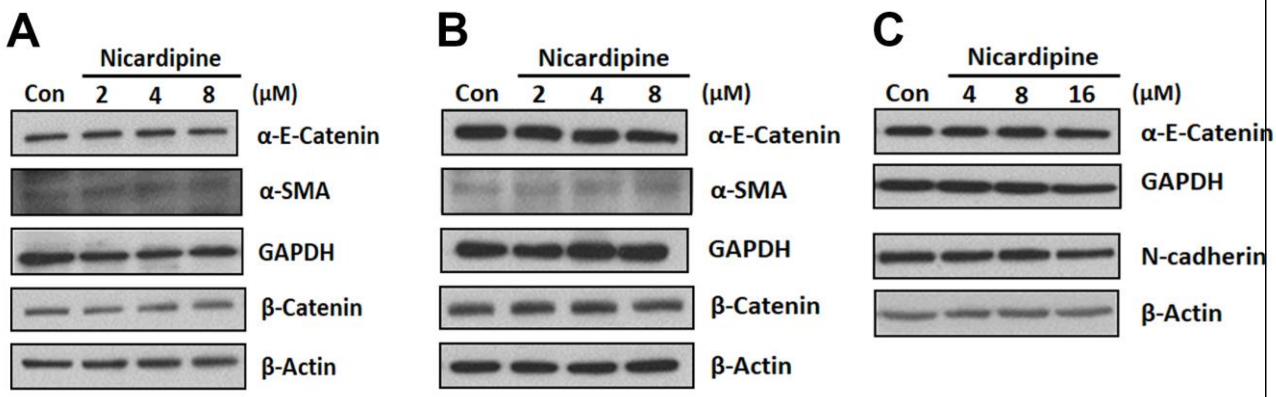

Supplementary Figure 2. The expression of markers of epithelial-mesenchymal transition on breast cancer cells. Under the treatment of nicardipine for 24 hours, the expression of epithelial and mesenchymal markers on MDA-MB-231 **(A)**, 4T1 **(B)**, and JC **(C)** cells were no significant influenced. Representative data from three independent experiments were shown here.
